# Intravoxel Incoherent Motion-Magnetic Resonance Imaging as an Early Predictor of Treatment Response to Neoadjuvant Chemotherapy in Locoregionally Advanced Nasopharyngeal Carcinoma: Erratum

**DOI:** 10.1097/01.md.0000469400.99238.dd

**Published:** 2015-07-02

In the article “Intravoxel Incoherent Motion-Magnetic Resonance Imaging as an Early Predictor of Treatment Response to Neoadjuvant Chemotherapy in Locoregionally Advanced Nasopharyngeal Carcinoma”,^1^ which appeared in Volume 94, Issue 24 of *Medicine*, the acknowledgments statement was incorrectly listed. The article has since been corrected online.
